# User-friendly microfluidic system reveals native-like morphological and transcriptomic phenotypes induced by shear stress in proximal tubule epithelium

**DOI:** 10.1063/5.0143614

**Published:** 2023-08-07

**Authors:** Natalie N. Khalil, Andrew P. Petersen, Cheng J. Song, Yibu Chen, Kaelyn Takamoto, Austin C. Kellogg, Elaine Zhelan Chen, Andrew P. McMahon, Megan L. McCain

**Affiliations:** 1 Alfred E. Mann Department of Biomedical Engineering, USC Viterbi School of Engineering, University of Southern California, Los Angeles, California 90089, USA; 2 Department of Stem Cell Biology and Regenerative Medicine, Keck School of Medicine of USC, University of Southern California, Los Angeles, California 90033, USA; 3 Eli and Edythe Broad Center for Regenerative Medicine and Stem Cell Research, Keck School of Medicine of USC, University of Southern California, Los Angeles, California 90033, USA; 4 Amgen Research, Cardiometabolic Disorders, San Francisco, California 94080, USA; 5 USC Libraries Bioinformatics Service, University of Southern California, Los Angeles, California 90089, USA

## Abstract

Drug-induced nephrotoxicity is a leading cause of drug attrition, partly due to the limited relevance of pre-clinical models of the proximal tubule. Culturing proximal tubule epithelial cells (PTECs) under fluid flow to mimic physiological shear stress has been shown to improve select phenotypes, but existing flow systems are expensive and difficult to implement by non-experts in microfluidics. Here, we designed and fabricated an accessible and modular flow system for culturing PTECs under physiological shear stress, which induced native-like cuboidal morphology, downregulated pathways associated with hypoxia, stress, and injury, and upregulated xenobiotic metabolism pathways. We also compared the expression profiles of shear-dependent genes in our *in vitro* PTEC tissues to that of *ex vivo* proximal tubules and observed stronger clustering between *ex vivo* proximal tubules and PTECs under physiological shear stress relative to PTECs under negligible shear stress. Together, these data illustrate the utility of our user-friendly flow system and highlight the role of shear stress in promoting native-like morphological and transcriptomic phenotypes in PTECs *in vitro*, which is critical for developing more relevant pre-clinical models of the proximal tubule for drug screening or disease modeling.

## INTRODUCTION

Drug-induced nephrotoxicity is a major cause of drug attrition during clinical trials.^1^ Human kidneys are subdivided into approximately one million nephrons, in which blood is first filtered as it travels from the glomerular capillaries to the Bowman's capsule, then subjected to reabsorption in the proximal tubule.^2,3^ The proximal tubule is the most metabolically active section of the nephron and a major site of drug accumulation. It also plays a major role in the secretion of xenobiotics, including prescription drugs,^4^ which are metabolized by cytochrome P450 family enzymes that may also generate toxic intermediate metabolites.^5^ As a result, the proximal tubule is highly susceptible to drug-induced toxicity, highlighting the need for predictive pre-clinical models of the human proximal tubule.^1^

Modeling the proximal tubule *in vitro* has conventionally been limited to static culture of primary or immortalized PTECs, which exhibit a flat, non-polarized architecture with limited functionality.^6,7^ However, *in vivo*, human PTECs are exposed to fluid flow that induces shear stresses between 0.3 and 1.2 dyn/cm^2^,^8^ inspiring the development of microfabricated systems to culture PTECs under flow *in vitro*. For example, PTECs have been cultured on a porous silicone membrane bonded between two microfluidic channels^9^ or around the lumen of three-dimensional (3D) channels printed within hydrogels.^10–16^ Exposing PTECs to physiological levels of shear stress using these systems has resulted in tissue polarization, including increased localization of laminin to the basement membrane^10–12^ and increased cell height.^9,10^ Physiological shear stress has also been shown to induce expression of important features of proximal tubules, including sodium–potassium pumps (Na/K-ATPase), which generate sodium gradients; aquaporin-1, which reabsorbs water; and primary cilia, which sense flow.^17^ However, existing flow systems are relatively expensive and/or difficult to implement by users that are inexperienced with microfabrication or microfluidics, which has limited their broad adoption by the research community. The impact of shear stress on the PTEC transcriptome has also not been fully characterized or compared to native proximal tubules, which is essential for evaluating the relevance of PTECs cultured under flow as a model for drug-induced nephrotoxicity and disease modeling.

Our goal was to engineer a relatively low-cost, user-friendly, and easy-to-customize system for exposing PTECs to fluid flow *in vitro* and then use it to evaluate shear-dependent changes in PTEC morphology and gene expression. Using a consumer-grade benchtop 3D extrusion printer and off-the-shelf materials, we designed and fabricated a flow circuit in which media travels from a syringe or peristaltic pump, through a custom bubble trap, into a commercial channel slide housing a monolayer of PTECs, and then into a custom media reservoir. We also laser-cut polypropylene sheets into an interlocking tray to hold and transport four flow circuits in parallel to easily accommodate media changes and imaging. Using this system, we cultured human PTECs under negligible or physiological levels of shear stress and evaluated cell phenotype through fluorescence microscopy and RNA sequencing. We observed that physiological shear stress promoted cuboidal-like cell morphology, downregulated pathways related to glycolysis, hypoxia, and wound healing, and upregulated xenobiotic drug metabolism pathways, which is especially relevant for drug screening. Together, these results validate our user-friendly flow system and reveal new changes in PTEC phenotype induced by physiological shear stress.

## RESULTS

### Design and fabrication of the microfluidic system

**FIG. 1.**
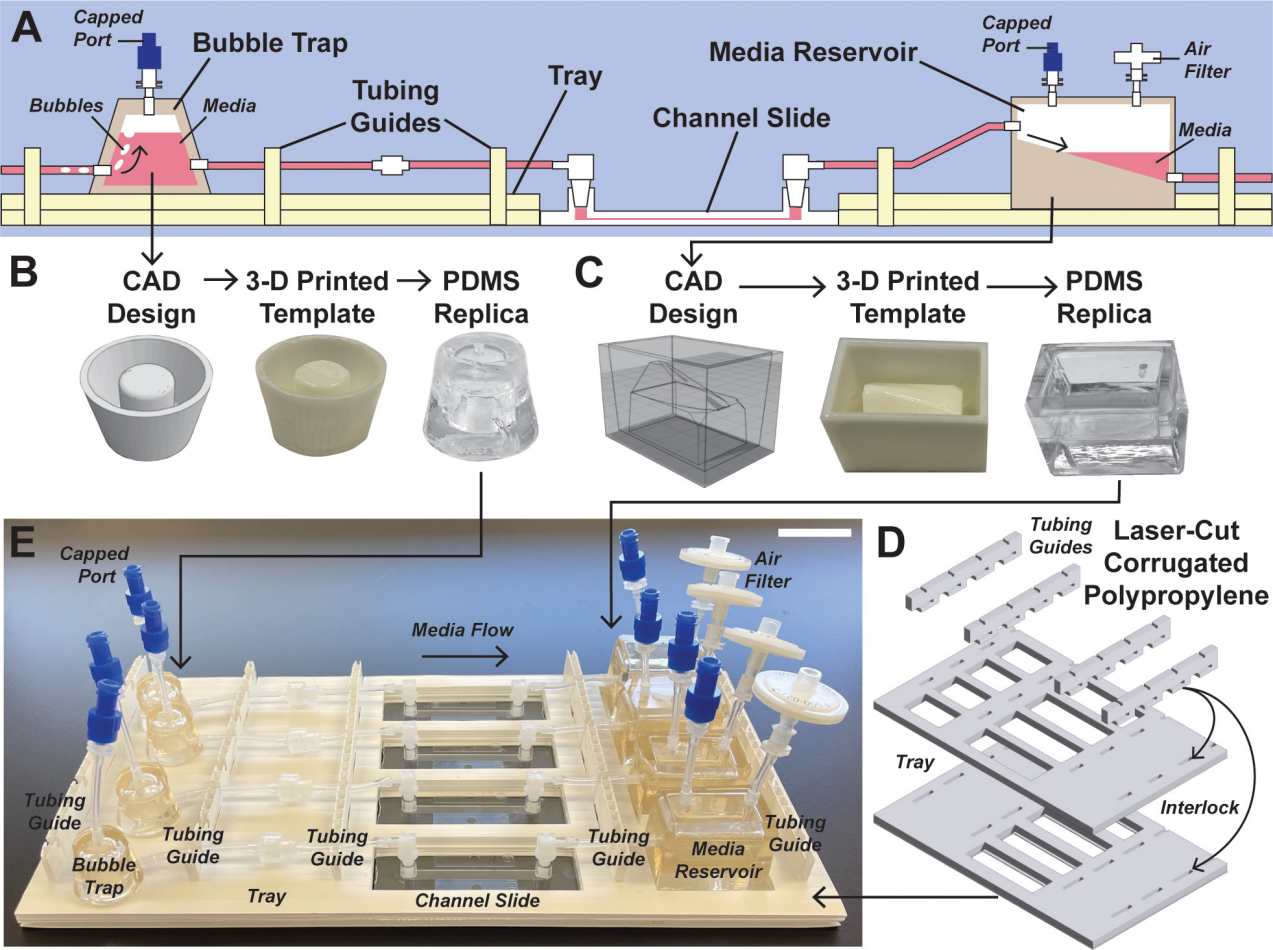
Design and fabrication of the microfluidic system. (a) Schematic of the assembled flow system. (b) Computer aided design (CAD), 3D-printed template, and PDMS replica of the bubble trap. (c) CAD, 3D-printed template, and PDMS replica of the media reservoir. (d) Schematic of the laser-cut tray and tubing guides, designed to hold four complete flow systems. (e) Photograph of an assembled flow system with four channel slides and all accessories. Scale bar: 1 in.

Our first goal was to develop a complete system for culturing human PTECs under physiological shear stress that is user-friendly to fabricate, assemble, operate, and customize. Thus, we selected equipment and materials that are inexpensive, off-the-shelf, and/or relatively commonplace in academic research labs. As shown in Fig. 1(a), one complete flow circuit consists of a custom bubble trap, a commercial channel slide, and a custom media reservoir. The bubble trap is a sealed reservoir with inlet and outlet ports at the bottom [Fig. 1(b)]. As media flows in from a syringe or peristaltic pump, air travels to the top of the reservoir and accumulates above the media. The trap can hold up to 1 ml of air before requiring manual venting by opening a capped port in the lid. Due to the relatively large volume of the bubble trap, manual venting should not be needed for experiments that last several days or potentially longer. The large volume of the bubble trap relative to anticipated bubble accumulation was primarily dictated by the relatively coarse resolution of 3D extrusion printing and PDMS replica molding, which was used for fabrication, as described below. A compact reservoir was designed to collect media exiting the channel slides [Fig. 1(c)]. The bottom of the reservoir is a shallow half-cone that funnels media to the outlet tubing, allowing low volumes of media to be recirculated without introducing bubbles. The reservoir lid houses a port connected to a sterile air filter to equalize the pressure inside the tubing with atmospheric pressure, which is critical for preventing pressure changes in the system caused by any movement of the components during routine tasks, such as imaging or changing media. Templates for the bubble trap and media reservoir [supplementary material, Figs. 1(a) and 1(b)] were designed using TinkerCAD (a free, browser-based computer aided design software) and fabricated using a consumer-grade 3D extrusion printer. PDMS reservoirs were replica-molded on the 3D-printed templates, sealed with a slab of PDMS as a lid, and biopsy punched to create inlet and outlet ports. We also 3D-printed tubing clips [supplementary material, Fig. 1(c)] to clamp the inlet and outlet of each channel slide when filling the flow system with media or removing the system for imaging to prevent exposing cells to abrupt changes in pressure.

A major challenge facing the broad adoption of microfluidic systems is the disorganization of tubing and flow components within a cell culture incubator, which can frustrate users and compromise experimental reproducibility. To address this, we designed a custom tray (L × W = 11.5 ×  6 in.^2^) to hold up to four channel slides and their accessories in parallel. As shown in Fig. 1(d), we laser-cut slots for slides and accessories into two sheets of corrugated polypropylene, which is readily available at consumer hardware stores and autoclavable. Five vertical tubing guides were similarly laser-cut and then interlocked into the two larger sheets to secure the layers together and provide notches to guide and stabilize tubing. Assembly instructions for the complete flow system are provided in Fig. 2 of the supplementary material. The tray enables a user to easily assemble four complete flow circuits [Fig. 1(e)] in a biological safety cabinet and transport them between a cell culture incubator and standard phase contrast microscope stage. The tray could also be used to immobilize components for live imaging experiments, although environmental conditions would need to be controlled by external equipment, such as a heated stage or microscope enclosure.

**FIG. 2.**
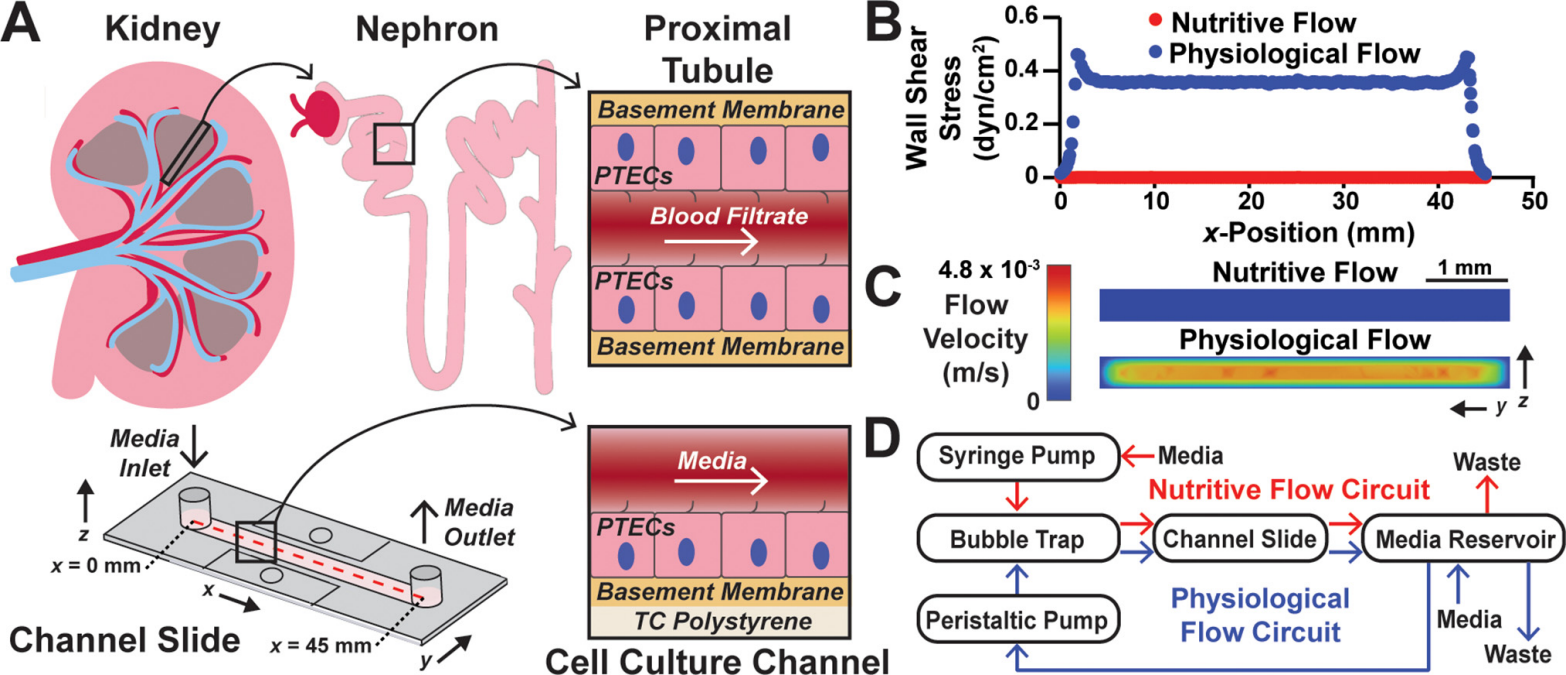
Simulation of shear stresses in the channel slide. (a) Schematic of unidirectional flow in the intact proximal tubule and in a monolayer of PTECs cultured in a channel slide. The fluid channel in the channel slide was approximated for shear stress simulations, where shear stress was calculated at each position along the dashed red line. (b) Simulations predict that a flow rate of 300 *μ*l/min (blue dots) induces a physiological shear stress of 0.35 dyn/cm^2^ over the centerline on the bottom surface of the chip. A flow rate of 1 ml/day (red dots) induces a nearly shear-less condition of 0.0008 dyn/cm^2^. (c) Flow velocity profiles for cross sections through the channel under nutritive and physiological flow. (d) Schematic of nutritive and physiological flow circuits.

In terms of cost, the only equipment required for fabricating a complete flow system are a consumer-grade 3D extrusion printer and laser cutter, which cost approximately $2000 and $10 000, respectively, and are very common in academic makerspaces. All materials (PDMS, polypropylene, tubing, etc.) needed for one complete system can be purchased for under $500. All components, except for the channel slides used for cell culture, are autoclavable and re-useable unless damaged. Users will also need to purchase a syringe and/or peristaltic pump to apply flow, which are available for under $1000. Cumulatively, these costs are well below the price points of commercial microfluidic systems.

### Simulation of shear stresses in the channel slide

We next used multiphysics simulation software to determine a volumetric flow rate that would generate shear stresses in the channel slides that are similar to those of native proximal tubules [Fig. 2(a)]. Using a “guess-and-check” approach, we determined that a flow rate of 300 *μ*l/min generated 0.35 dyn/cm^2^ of shear stress on the bottom of the channel slide [Figs. 2(b) and 2(c)]. This condition was termed “physiological flow” since it is within the physiological range of 0.3–1.2 dyn/cm^2^.^8^ Due to the large volumes of media required at this flow rate, we recirculated the media using a peristaltic pump [Fig. 2(d)]. We also applied 1 ml/day as a nearly shear-less control condition (shear stress calculated as 0.0008 dyn/cm^2^), termed “nutritive flow,” using a syringe pump [Figs. 2(b) and 2(c)]. In this condition, media was collected in the reservoir and then discarded instead of being recirculated due to the low volume demands.

### Physiological shear stress promotes native-like PTEC morphology

**FIG. 3.**
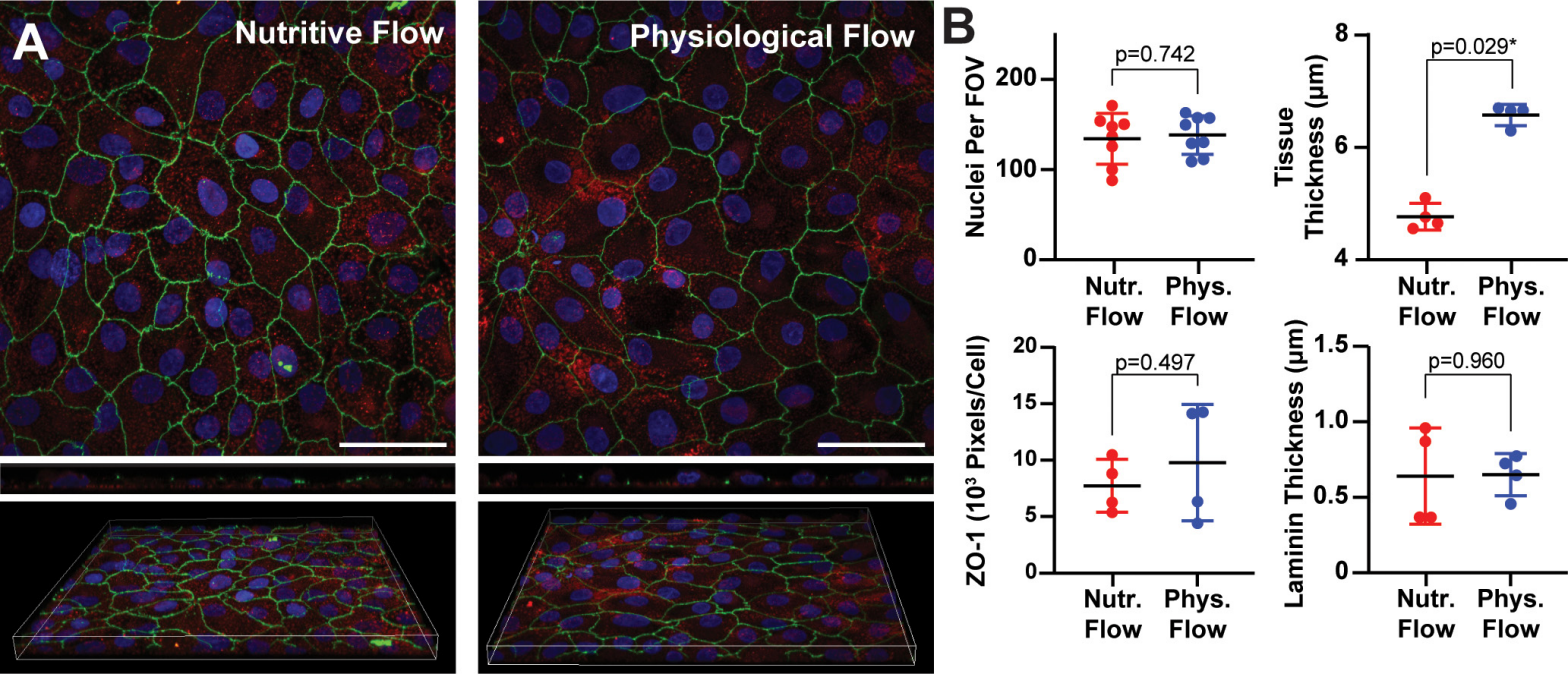
Shear-dependent changes in nuclei, ZO-1, and tissue thickness. (a) PTECs stained for laminin (red), ZO-1 (green), and DAPI (blue) after exposure to three days of nutritive or physiological flow. Scale bar: 50 *μ*m. In bottom row, width × depth × height of white box = 212.13 × 212.13 × 12.8 *μ*m^3^ (left) or 212.13 × 212.13 × 10 *μ*m^3^ (right). (b) Number of nuclei per field of view (FOV), normalized ZO-1 pixel count, tissue thickness, and laminin thickness, calculated from confocal z-stacks. P-value indicates results of unpaired t-test (nuclei, ZO-1, laminin) or Mann–Whitney test (tissue thickness). ^*^indicates p-value < 0.05.

Next, we cultured immortalized human PTECs in channel slides and used our system to apply physiological or nutritive flow for three days. To assess apical-basal polarity, we stained tissues for ZO-1, laminin, and nuclei and collected confocal z-stacks. As expected for PTECs, ZO-1 localized to tight junctions in a cobblestone pattern and laminin localized to the basal surface in all conditions [Fig. 3(a)]. The number of nuclei and 3D coverage of ZO-1 [Fig. 3(b)] were similar between flow conditions (p = 0.7422 and 0.4968, respectively), suggesting that cell viability and cell–matrix and cell–cell adhesions were not strongly affected by flow. Previous studies using cell lines have also reported that ZO-1 expression is not affected by similar levels of shear stress in PTECs.^8,9,18^ However, studies with primary cells have reported increased ZO-1 staining in response to flow.^19^ Thus, tight junction formation in cell lines may be less sensitive to flow compared to primary cells, potentially due to more phenotypic drift in cell lines. Tissue thickness, as measured from composite images of ZO-1, nuclei, and laminin to account for both cells and the extracellular matrix, was significantly higher under physiological flow (p = 0.0286), while laminin thickness was conserved (p = 0.9601) [Fig. 3(b)]. This suggests that physiological flow induced an increase in cell height, consistent with a more cuboidal phenotype, similar to previous findings.^9,10^

**FIG. 4.**
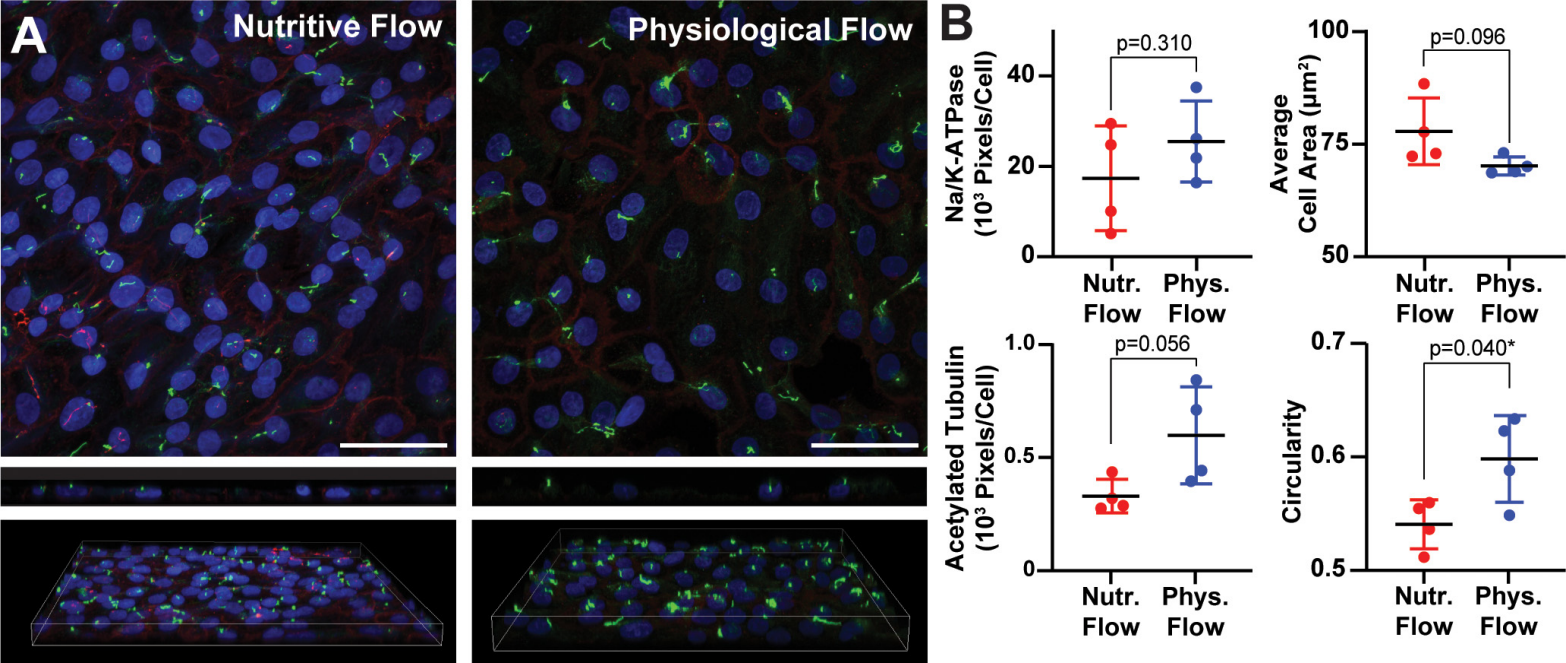
Shear-dependent changes in Na/K-ATPase, cilia, and cell geometry. (a) PTECs stained for Na/K-ATPase (red), acetylated tubulin (green), and DAPI (blue) after exposure to three days of nutritive or physiological flow. Scale bar: 50 *μ*m. In bottom row, width × depth × height of white box = 212.13 × 212.13 × 11.40 *μ*m^3^ (left) or 212.13 × 212.13 × 18 *μ*m^3^ (right). (b) Normalized Na/K-ATPase pixel count, normalized acetylated tubulin pixel count, average cell area, and cell circularity, calculated from confocal z-stacks and analyzed with unpaired t-test. ^*^indicates p-value < 0.05.

We next stained tissues for acetylated tubulin to visualize primary cilia and Na/K-ATPase [Fig. 4(a)]. In both conditions, we observed basolateral expression of Na/K-ATPase and primary cilia protruding from the apical surface, as expected. Similar to previous studies,^9,20,21^ the 3D coverage of Na/K-ATPase (p = 0.3036) [Fig. 4(b)], the 3D coverage of acetylated tubulin (p = 0.0558), and the percentage of ciliated cells (p = 0.1814) [supplementary material, Fig. 3(b)] trended higher with physiological flow, but did not reach statistical significance. The lack of statistical significance could be partially attributed to the variability across experiments, which was also generally higher in physiological flow compared to nutritive flow. This variability may be attributed to the inherent variability of immunostaining assays as well as the use of a cell line, for which some cells may be more or less sensitive to flow due to the phenotypic drift caused by the immortalization process and repeated passaging. We also measured cilia length from 3D z-stacks, as some studies have shown increased cilia length with flow,^22^ but we did not observe differences in cilia length (p = 0.6857) (supplementary material, Fig. 4). From the Na/K-ATPase images, we also characterized cell shape and found a significant increase in circularity (p = 0.0396) and a slight decrease in surface area at the apical surface (p = 0.09) [Fig. 4(b)], suggestive of a shift toward native-like cuboidal cell morphology with shear stress.

### Physiological shear stress suppresses injury pathways and increases drug metabolism genes and pathways

**FIG. 5.**
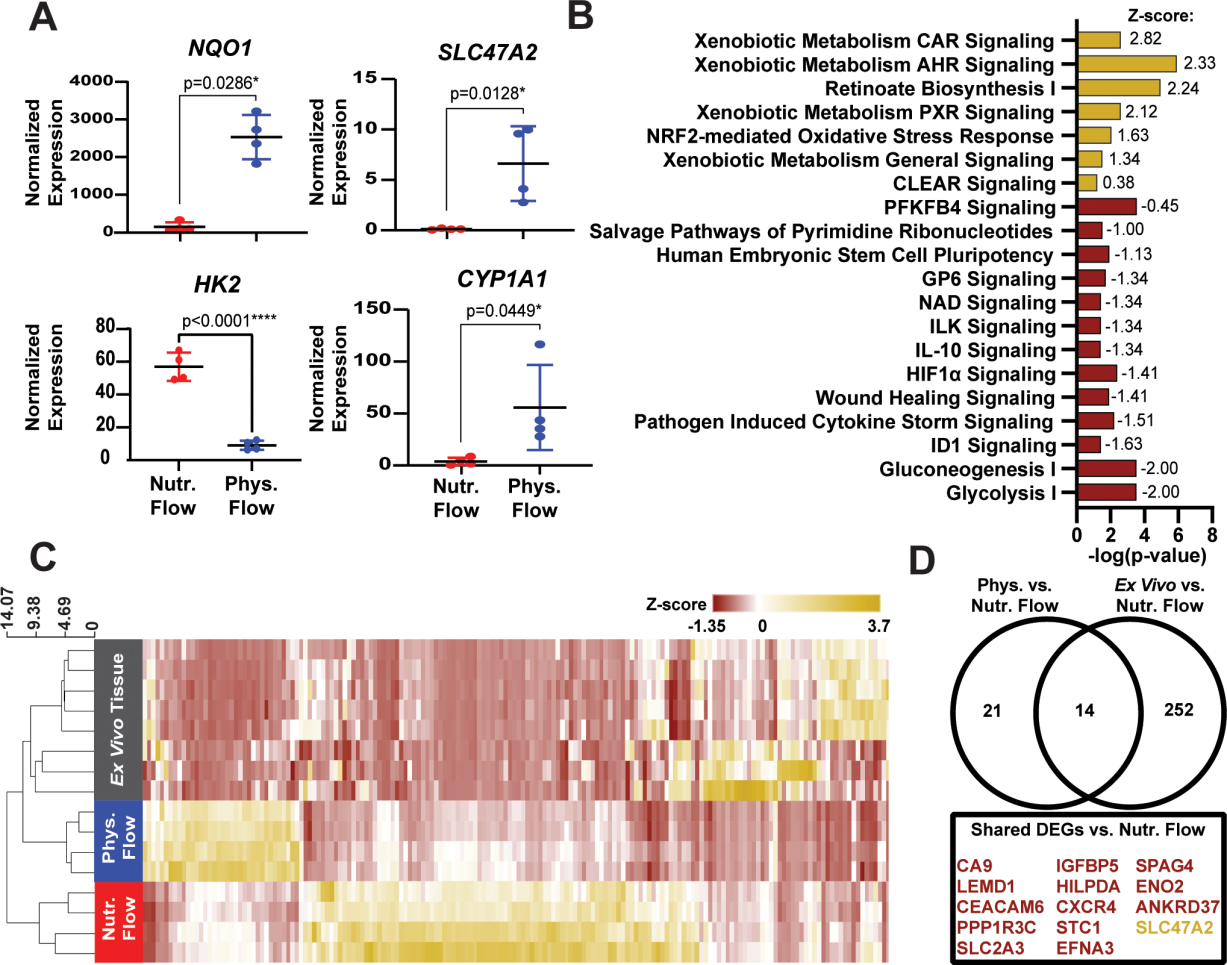
Shear-dependent changes in PTEC transcriptome. (a) Relative expression of *NQO1*, *SLC47A2*, *HK2* and *CYP1A1* in physiological vs nutritive flow. P-value indicates results of Mann-Whitney Test (*NQO1*) or unpaired t-test (*SLC47A2*, *CYP1A1*, and *HK2*). ^*^indicates p-value < 0.05. (b) Pathways enriched by physiological flow, with p-value < 0.05, when compared with nutritive flow. Z-score is annotated and suggests pathways that are up-regulated (yellow, positive z-score) or downregulated (red, negative z-score) by physiological flow. (c) Heat map and hierarchical clustering of 343 DEGs between PTECs cultured under physiological vs nutritive flow, compared with *ex vivo* tissue. (d) Venn diagram comparing the 343 shear-dependent genes after differential expression analysis of physiological vs nutritive flow and *ex vivo* vs nutritive flow.

To identify shear-mediated changes in the transcriptome, we performed RNA sequencing on PTECs cultured with nutritive or physiological flow for three days. Differential expression analysis (FDR ≤ 0.05) identified 343 differentially expressed genes (DEGs) between physiological vs nutritive flow (supplementary material, Table I). Similar to previous studies in vascular endothelial cells^23–25^ and PTECs,^26^ physiological flow upregulated NAD(P)H Quinone Dehydrogenase 1 (*NQO1*) (p = 0.0286), which is involved in oxidative stress handling [Fig. 5(a)]. Relatedly, pathway analysis (supplementary material, Table II) demonstrated upregulation of the Nrf2 mediated oxidative stress response pathway [Fig. 5(b)], similar to previous work.^26^ This may be due to increased accumulation of metabolites in recirculated media, such as reactive oxygen species that are normal byproducts of cellular activity. Physiological shear stress also upregulated *SLC47A2*, which encodes MATE-2K, a cationic drug transporter (p = 0.0128) [Fig. 5(a)], as reported previously,^26^ and *CYP1A1* (p = 0.0449) [Fig. 5(a)], a member of the cytochrome P450 family of enzymes.^5^ Our pathway analysis also revealed upregulation of three pathways of xenobiotic metabolism: signaling by the aryl hydrocarbon receptor (AHR), pregnane X receptor (PXR), and constitutive androstane receptor (CAR), as well as the xenobiotic metabolism general signaling pathway [Fig. 5(b)]. AHR, PXR, and CAR are nuclear receptors that, upon binding to xenobiotics, act as transcription factors for target genes, including drug-metabolizing enzymes and drug transporters that detoxify and eliminate xenobiotics from the body.^27,28^ These shear-induced increases in *SLC47A2*, *CYP1A1*, and xenobiotic metabolism signaling pathways may have an especially critical impact for screening drug-induced nephrotoxicities *in vitro*. Gene specific analysis also showed downregulation of *HK2*, a marker of glycolysis [Fig. 5(a)]. Consistent with this, pathway analysis showed downregulation of glycolysis and hypoxia-inducible factor (HIF) signaling pathways in response to physiological flow [Fig. 5(b)]. This may be attributed to increased availability of oxygen with increased flow rate and/or decreased availability of glucose in spent media. We also observed downregulation of pathways for wound healing signaling, IL-10 signaling, and cytokine storm signaling, suggesting that physiological flow suppresses select inflammation and injury pathways commonly observed in static PTEC cultures [Fig. 5(b)].^29^

### Physiological shear stress promotes transcriptional profiles similar to intact proximal tubules

Finally, we asked how the transcriptome of our *in vitro* tissues compared to that of intact proximal tubule tissue. To answer this, we compared the expression levels of the 343 DEGs identified between physiological and nutritive flow with a publicly available dataset of proximal tubule segments isolated from healthy human donors.^30,31^ As shown by the dendogram in Fig. 5(c), the *in vitro* tissue with physiological flow clustered more closely with the *ex vivo* tissue compared to the nutritive flow condition, suggesting that physiological shear stress induced changes in gene expression that shifted cells closer to the phenotype of endogenous proximal tubules.

We next normalized samples from all three conditions together, performed differential expression analysis of physiological flow vs nutritive flow and *ex vivo* tissue vs nutritive flow, and filtered the resulting list of DEGs to the 343 shear-dependent genes identified previously. Due to the expected wide variability between *in vitro* and *ex vivo* tissues, we relaxed the FDR cutoff to 0.1 for this comparison, resulting in 252 DEGs in *ex vivo* vs nutritive flow and 21 DEGs in physiological vs nutritive flow. We then generated a Venn diagram comparing the resulting shear-dependent genes and identified 14 genes that trended in the same direction (i.e., upregulated or downregulated) in both physiological flow and *ex vivo* tissue when compared with nutritive flow [Fig. 5(d)]. Most genes in this category were downregulated in physiological flow and *ex vivo* tissue but upregulated in nutritive flow, including *ENO2*, a glycolytic enzyme;^32^ *HILPDA* and *SLC2A3*, which are both activated by hypoxia;^33,34^ and *CXCR4*, which is upregulated after injury.^35^ These observations further suggest that, compared to PTECs cultured with negligible levels of shear stress, PTECs cultured with physiological shear stress have a transcriptional phenotype that is both healthier and more consistent with intact proximal tubules.

## DISCUSSION

Applying shear stress via microfluidic systems has been shown to enhance the physiological phenotype of PTECs *in vitro*^9,10,22,26^ and other cell types exposed to fluid flow *in vivo.*^23–25^ However, most existing flow systems are relatively expensive and/or complex to setup and use. Here, we describe the development and implementation of a microfluidic system that is simple to manufacture, inexpensive, and easy to operate and customize, which can empower non-experts or resource-limited researchers to adopt microfluidic devices into their workflow. Although we tailored our system to four channel slides, the modular components can be easily modified for different quantities or types of microfluidic devices for a broad range of applications in biomedical research or pre-clinical drug screening.

Using our system, we found that PTECs exposed to nutritive or physiological flow demonstrated apical-basal cell polarity with similar expression of ZO-1 and laminin. However, PTECs under physiological flow trended toward increased cilia and Na/K-ATPase coverage and a more native-like cuboidal morphology, consistent with previous studies.^9,10^ Our transcriptomic analysis revealed that physiological flow also downregulated several stress or injury pathways that are commonly expressed in static cultures^29^ and suppressed glycolysis and HIF1α signaling, suggestive of a healthier metabolic phenotype. In relation to drug metabolism, physiological flow upregulated a cationic drug transporter (*SLC47A2*), a drug metabolizing enzyme (*CYP1A1*), and several pathways associated with xenobiotic metabolism (PXR, CAR, AHR, and General Signaling). Because the proximal tubule is a major route for the metabolism and excretion of drugs,^4^ the expression of these genes and pathways likely has a substantial impact on the ability of PTECs to accurately predict drug metabolism, toxicity, and efficacy *in vitro*. Consistent with our findings, other engineered proximal tubule models have demonstrated improved sensitivity to known nephrotoxins, such as cisplatin^5,9^ and cyclosporine A,^10^ with flow. Similar evaluation of drug responses in our system under a range of shear stresses is an important follow-up study. Finally, when compared to *ex vivo* proximal tubule tissue, genes that were differentially expressed between physiological flow and nutritive flow clustered physiological flow with *ex vivo* tissue, indicating that culturing PTECs with physiological shear stress induces transcriptional changes that are consistent with intact proximal tubules. Collectively, these data suggest that physiological shear stress induced more native-like phenotypes in PTECs on morphological and transcriptional levels, reinforcing that shear stress is an especially key feature for improving the relevance of PTECs as a model for *in vitro* drug screening or disease modeling.

In this study, we used an immortalized cell line as a simple approach for validating our flow system.^36,37^ Although these cells were simple to acquire and use, we did observe dampened and more variable responses to flow compared to primary cells, indicating that they may not have sufficiently relevant phenotypes for certain applications. Furthermore, PTECs from different regions of the proximal tubule have slightly different phenotypes^38^ and likely experience slightly different shear stresses, but we neglected to account for the regional specificity of the proximal tubule for this study. However, our system should also be compatible with primary^39^ or human induced pluripotent stem cell (hiPSC)-derived kidney cells,^40,41^ including those derived from organoids.^42,43^ Our system would also be compatible with microfluidic devices that house 3D constructs, such as entire organoids^21^ or tissue explants.^44^ Based on our findings and others,^43^ we would expect that each of these cell or tissue sources would express more physiologically relevant phenotypes under shear stress. Thus, combining our user-friendly flow system with patient-derived cells or tissues could establish more meaningful human genotype-phenotype relationships in the context of disease modeling and more accurate patient-specific drug responses. Changes in shear stress also occur in certain types of kidney disease.^45^ For example, in diseases with loss of glomeruli or tubules, nephrons may compensate through tubular dilation, obstruction, or hyperfiltration, resulting in supraphysiological shear stresses,^20,46^ which could also be modeled in our system. More broadly, many anatomical and physiological features, such as tubule diameter, blood pressure, and tubule length, can also affect flow patterns and shear stresses and likely also interact with patient-specific genotypic features. Thus, our system could be extended to systematically investigate a plethora of both genetic and biomechanical causes of pathological remodeling in the proximal tubule.

In summary, we developed a complete flow system for culturing human PTECs under defined shear stress using off-the-shelf materials and benchtop fabrication equipment to reduce the “barrier to entry” for non-experts. We then used our system to reveal new shear-dependent phenotypic changes in PTECs that mimic intact proximal tubules and are especially relevant to drug metabolism. This system can be further improved by integrating more relevant human cell types and developing more sophisticated functional readouts, such as glucose reabsorption.

## METHODS

### Multiphysics modeling of shear stress

Ansys Fluent (Ansys, Inc., Canonsburg, PA) was used to create a three-dimensional (3D) approximation of the fluid path within a channel slide (ibidi *μ*-Slide I, 0.4 Luer; ibidi GmbH, Gräfelfing, Germany). The fluid path was modeled as an elliptical channel with 0.4 mm height, 5 mm width, and 50 mm length with cylindrical inlets and outlets of diameter 3.5 mm and height 5.8 mm. After setting the initial and boundary conditions, the software was used to solve governing conservation of mass and momentum equations to calculate shear stress based on inlet velocity.^47^ Shear stress was estimated along a centerline at the bottom surface of the channel, where cells would attach. A guess-and-check approach was used to identify a flow rate and corresponding inlet velocity that generated shear stress in the physiological range of 0.3 and 1.2 dyn/cm^2^ (Ref. 8). Ultimately, a flow rate of 300 *μ*l/min, corresponding to an inlet velocity of 5.2 × 10^−4^ m/s, was chosen, which generates 0.35 dyn/cm^2^ of shear stress. The nutritive flow rate of 1 ml/day was chosen empirically and corresponded to a velocity of 1.2 × 10^−8^ m/s and shear stress of 0.0008 dyn/cm^2^. Cell media was approximated to have the viscosity and density of water, similar to previous work.^48^

### Flow system fabrication

#### Laser-cut polypropylene platform

The interlocking tray [supplementary material, Fig. 1(d)] was designed in CorelDRAW X7 (Corel Corporation, Ottawa, Canada) and cut into corrugated polypropylene (Plaskolite White Corrugated Plastic Sheets, Lowe's Corporate, Mooresville, NC, USA) with an Epilog Mini 18 laser-cutter (Epilog Laser, Golden, CO, USA). Corrugated polypropylene consists of two horizontal layers connected with vertical corrugations. For full cuts through a corrugated sheet, we used power, speed, and frequency settings of 93, 32, and 5000, printing 10 copies. For partial cuts through just one horizontal layer, we used power, speed, and frequency settings of 93, 60, and 5000, printing 1 copy.

#### Custom PDMS components

Templates for bubble traps and media reservoirs [supplementary material, Figs. 1(a) and 1(b)] were designed in TinkerCAD (Autodesk, Inc., San Rafael, CA, USA) and are publicly available for download on GitHub,^49^ 3D-printed in acrylonitrile butadiene styrene (ABS) using a MakerBot Replicator 2X Experimental 3D Printer (Makerbot Industries, New York, NY, USA), and smoothed by immersing in acetone then drying for 24 h. Polydimethylsiloxane (PDMS) was made by mixing elastomer base and curing agent in a 10:1 (w/w) ratio (PDMS, Sylgard 184; Dow Corning Corporation, Midland, MI, USA) using a planetary centrifugal Thinky Mixer AR-100 (Thinky Corporation, Tokyo, Japan). PDMS was poured onto the 3D-printed ABS templates, cured in a 65 °C oven for at least 4 h, removed from the templates, and biopsy punched to create 2 mm diameter holes for tubing inlets and outlets. PDMS chambers were enclosed with a PDMS lid by painting the exposed surface with uncured PDMS, placing in contact with a 4–5 mm slab of PDMS, and then curing together at 65 °C for another 4 h.

#### Flow system design and assembly

Silicone tubing with barbed and luer plastic connectors and caps were used to connect components and are listed in the supplementary material, Fig. 2 (ibidi GmbH, Gräfelfing, Germany). Before assembly, all individual components were washed with 10% bleach and rinsed with DI water. Bubble traps and media reservoirs were then fully assembled and autoclaved with a standard dry goods cycle. In a biological safety cabinet, the polypropylene tray was assembled with all components and tubing. The step-by-step assembly process is shown in the supplementary material, Fig. 2.

Media flow was applied by a 6-channel NE-1600 syringe pump (New Era Pump Systems, Inc., Farmingdale, NY, USA) or an Ismatec 8-channel digital peristaltic pump (Cole-Parmer, Vernon Hills, IL, USA) with two-stop, 1.85 mm ID, silicone peroxide tubing. Media travels from the syringe or peristaltic pump through the bubble trap, channel slides, and media reservoir with 0.4 *μ*m pore sterile air filter (Drummond Scientific Company, Broomall, PA, USA).

### Cell culture and exposure to fluidic shear-stress

Human hTERT-immortalized PTECs (ATCC, Manassas, VA, USA) were cultured in DMEM:F12 (ATCC, Manassas, VA, USA) supplemented with the hTERT immortalized RPTEC growth kit (ATCC, Manassas, VA, USA) and 1% fetal bovine serum (FBS) in 75 cm^2^ tissue culture flasks. Once confluent, cells were dissociated with TrypLE Express (Thermo Fisher Scientific, Waltham, MA, USA), counted, and seeded onto ibidi channel slides (ibidi *μ*-Slide I, ibiTreat, 0.4 mm channel height; ibidi GmbH, Gräfelfing, Germany) at a density of 200,000 cells/cm^2^ and then incubated at 37 °C for 5.5 h until adherent. Channel slides were then inserted into the flow system and connected to a 6-channel NE-1600 syringe pump (New Era Pump Systems, Inc., Farmingdale, NY, USA) to receive 1 ml/day (nutritive flow) until confluent. Prior to starting media flow, 3D-printed ABS tubing clips [supplementary material, Fig. 1(c)] were attached to the inlet and outlet tubing of channel slides to prevent exposure of cells to abrupt changes in pressure. Once confluent, cells were serum-arrested and then either continued to receive 1 ml/day (nutritive flow) from the syringe pump or were switched to an Ismatec 8-channel digital peristaltic pump (Cole-Parmer, Vernon Hills, IL, USA) with two-stop, 1.85 mm ID, silicone peroxide tubing to receive 300 *μ*l/min (physiological flow) for 3 days. For physiological flow, the tubing was also rearranged to recirculate media from the reservoir back into the pump (supplementary material, Fig. 2).

### Immunofluorescence and imaging

Cells were washed with PBS, fixed with 4% paraformaldehyde for 10 min, then permeabilized and blocked with 0.25% Triton-X 100 and 2% Seablock for an additional 20 min. Cells were incubated with primary antibodies overnight at 4 °C, rinsed with 2% Seablock, then incubated with the corresponding secondary antibodies for 1 h at room temperature (antibodies listed in the supplementary material, Table III). For each chip, five widefield images were collected on a 60× oil objective with a Nikon Eclipse Ti-S inverted fluorescent microscope. Additionally, five confocal z-stacks were collected with a maximum step size of 0.4 *μ*m on a 60× oil objective with a Nikon C2 point-scanning confocal microscope.

### Image analysis

Widefield 60× images of tissues stained for DAPI and Na/K-ATPase were analyzed for cell area and cell circularity through custom ImageJ scripts implementing the MorphoLibJ plugin,^50^ which segments an image based on pre-defined markers [supplementary material, Fig. 3(a)]. Markers for each cell were identified by using the find maxima function on the DAPI stain. Then, the Na/K-ATPase stain was segmented using MorphoLibJ marker-controlled segmentation. The segmented image was then analyzed for apical circularity and cell area using the MorphoLibJ analyze particles function with an area exclusion filter. The values from five images per chip were averaged and reported as n = 1.

Widefield 60× images of tissues stained for DAPI and acetylated tubulin were analyzed to quantify the percentage of ciliated cells [supplementary material, Fig. 3(b)]. In ImageJ, the find maxima function was used to count the number of cilia and the analyze particles function was used to count nuclei. The percentage of ciliated cells was defined as the number of cilia normalized to the number of cells. The values from five images per chip were averaged and reported as n = 1.

Confocal 60× z-stacks were analyzed to approximate tissue thickness and laminin layer thickness through custom ImageJ scripts [supplementary material, Fig. 4(a)]. To measure tissue thickness, the projection of the orthogonal view of a z-stack composite of ZO-1, laminin, and nuclei was generated, binarized, and rotated 90° into vertical format. Ten pre-defined regions of interest were drawn onto the binarized projection. The plot profile function was then used to determine the number of non-zero pixels, which was converted into *μ*m and used to approximate tissue thickness. For each z-stack, ten regions of interest were averaged. Five z-stacks per chip were then averaged and reported as n = 1. The same process was performed on the laminin stain alone to measure laminin thickness.

To measure cilia length [supplementary material, Fig. 4(b)], the projections of orthogonal views of a z-stack of Na/K-ATPase, acetylated tubulin, and nuclei were generated and each channel was binarized. The nuclei and Na/K-ATPase signal was then subtracted from the acetylated tubulin signal such that only “apical” acetylated tubulin remained, which corresponds to cilia protruding from the cell. The plot profile function was then used to compute maximum cilia length, defined as the difference between the maximum and minimum distance between non-zero values in the plot profile. Five z-stacks per chip were averaged and reported as n = 1.

To determine the 3D coverage of ZO-1, acetylated tubulin, and Na/K-ATPase [supplementary material, Fig. 4(c)], a slice near the center of each z-stack was selected and the make binary function was then applied, using the default threshold. This same threshold was then applied to every slice to binarize the entire z-stack. A histogram of the binarized z-stack was then used to determine the total number of pixels above the threshold. The number of pixels was then normalized to the total number of nuclei in the stack, determined by the analyze particles function. Five z-stacks per chip were averaged and reported as n = 1.

Spreadsheets containing plot profile or histogram data from ImageJ scripts were sorted and analyzed through custom python scripts implemented in Brev.dev, a browser-based developer environment (San Francisco, CA, USA). All image analysis scripts are publicly available on GitHub.^49^

### RNA sequencing and data analysis

Cells were immediately lysed with 1% beta mercaptoethanol in Buffer RLT lysis buffer (QIAGEN, Hilden, Germany). A QIAGEN RNeasy Micro kit (QIAGEN, Hilden, Germany) was used to isolate RNA. RNA concentrations and QC were measured on Agilent TapeStation Systems (Agilent). RNA with RINe above 8.0 were submitted for sequencing at the Genome Technology Access Center at Washington University in St. Louis. Paired-end libraries were prepared using the Clontech SMARTer Illumina Kit (Illumina, San Diego, CA) and sequenced on a NovoSeq 6000 (Illumina, San Diego, CA) for 125 cycles. Sequences were trimmed from both ends based on a Phred quality score of 20 and a minimum read length of 25, aligned to the hg38 genome build using STAR (2.7.8a), and quantified using Gencode 38 (V2) annotation in Partek Flow Genomics Analysis software (Partek Inc., St. Louis, MO, USA). Read counts per gene were normalized to fragments per kilobase of transcript per million mapped reads (FPKM) and then analyzed for differential gene expression using Partek Gene Specific Analysis method with fold change and FDR cutoffs of 1.5 and 0.05, respectively. Finally, Ingenuity Pathway Analysis (QIAGEN, Hilden, Germany) software was used to perform pathway analysis using the fold change of each differentially expressed gene, with fold change and FDR cutoffs of 1.5 and 0.05, respectively. A comparison was made with the publicly available dataset in NCBI's Gene Expression Omnibus, GEO Series Accession No. GSE163603 at https://www.ncbi.nlm.nih.gov/geo/query/acc.cgi?acc=GSE163603.^30,31^ For this comparison, publicly available reads were imported into Partek Flow, subsampled to normalize the sequencing depth (supplementary material, Table IV), and re-analyzed together with our data using the above workflow. Our data were made publicly available in NCBI's Gene Expression Omnibus (GEO Series Accession No. GSE221871 at https://www.ncbi.nlm.nih.gov/geo/query/acc.cgi?acc=GSE221871).^51^

### Statistics

For imaging data, each datapoint was obtained by averaging five images per chip, collected across four independent trials. Data were then tested for normality using the Kolmogorov–Smirnov test and analyzed using unpaired t-tests or the Mann–Whitney test in GraphPad Prism (La Jolla, CA, USA). For RNA sequencing data, tissues were lysed from four chips collected over four independent trials.

## SUPPLEMENTARY MATERIAL

The supplementary material contains in-depth assembly instructions, image analysis demonstrations, and gene expression fold-change data.

## Data Availability

The data that support the findings of this study are openly available in NCBI's Gene Expression Omnibus at https://www.ncbi.nlm.nih.gov/geo/query/acc.cgi?acc=GSE221871, Ref. 51.
